# Late-Pregnancy Fetal Hypoxia Is Associated With Altered Glucose Metabolism and Adiposity in Young Adult Offspring of Women With Type 1 Diabetes

**DOI:** 10.3389/fendo.2021.738570

**Published:** 2021-10-27

**Authors:** Miira M. Klemetti, Kari Teramo, Hannu Kautiainen, Niko Wasenius, Johan G. Eriksson, Merja K. Laine

**Affiliations:** ^1^Obstetrics and Gynecology, University of Helsinki and Helsinki University Hospital, Helsinki, Finland; ^2^Lunenfeld-Tanenbaum Research Institute, Mount Sinai Hospital, Toronto, ON, Canada; ^3^Department of Medical Genetics, University of Helsinki and Helsinki University Hospital, Helsinki, Finland; ^4^Department of Obstetrics and Gynecology, South Karelia Central Hospital, Lappeenranta, Finland; ^5^Folkhälsan Research Center, Helsinki, Finland; ^6^Primary Health Care Unit, Kuopio University Hospital, Kuopio, Finland; ^7^National University of Singapore, Yong Loo Lin School of Medicine, Department of Obstetrics and Gynaecology and Human Potential Translational Research Programme, Singapore, Singapore; ^8^Singapore Institute for Clinical Sciences (SICS), Agency for Science, Technology and Research (A*STAR), Singapore, Singapore

**Keywords:** pregnancy, type 1 diabetes, hypoxia, erythropoietin, insulin, adiposity

## Abstract

**Objective:**

To investigate associations between exposure to fetal hypoxia and indicators of metabolic health in young adult offspring of women with type 1 diabetes (OT1D).

**Methods:**

156 OT1D born between 7/1995 and 12/2000 at Helsinki University Hospital, Finland, were invited for follow-up between 3/2019 and 11/2019. A control group of 442 adults born from non-diabetic pregnancies, matched for date and place of birth, was obtained from the Finnish Medical Birth Register. In total, 58 OT1D and 86 controls agreed to participate. All OT1D had amniotic fluid (AF) sampled for erythropoietin (EPO) measurement within two days before delivery in order to diagnose fetal hypoxia. In total, 29 OTID had an AF EPO concentration <14.0 mU/l, defined as normal, and were categorized into the low EPO (L-EPO) group. The remaining 29 OT1D had AF EPO ≥14.0 mU/ml, defined as fetal hypoxia, and were categorized into the high EPO (H-EPO) group. At the age of 18-23 years, participants underwent a 2-h 75g oral glucose tolerance test (OGTT) in addition to height, weight, waist circumference, body composition, blood pressure, HbA_1c_, cholesterol, triglyceride, high-sensitivity CRP and leisure-time physical activity measurements.

**Results:**

Two OT1D were diagnosed with diabetes and excluded from further analyses. At young adult age, OT1D in the H-EPO group had a higher BMI than those in the L-EPO group. In addition, among female participants, waist circumference and body fat percentage were highest in the H-EPO group. In the OGTTs, the mean (SD) 2-h post-load plasma glucose (mmol/L) was higher in the H-EPO [6.50 (2.11)] than in the L-EPO [5.21 (1.10)] or control [5.67 (1.48)] offspring (p=0.009). AF EPO concentrations correlated positively with 2-h post-load plasma glucose [r=0.35 (95% CI: 0.07 to 0.62)] and serum insulin [r=0.44 (95% CI: 0.14 to 0.69)] concentrations, even after adjusting for maternal BMI, birth weight z-score, gestational age at birth and adult BMI. Control, L-EPO and H-EPO groups did not differ with regards to other assessed parameters.

**Conclusions:**

High AF EPO concentrations in late pregnancy, indicating fetal hypoxia, are associated with increased adiposity and elevated post-load glucose and insulin concentrations in young adult OT1D.

## Introduction

Maternal type 1 diabetes increases the risk of adverse perinatal outcomes such as preterm birth, fetal macrosomia and intrauterine hypoxia (1–3). Fetal exposure to maternal diabetes may also predispose to neonatal complications (1) and have long-term effects on the offspring’s metabolism and cardiovascular function (4).

In pregnancies affected by maternal pre-existing diabetes, the risk of fetal death is ~4-5-fold compared to non-diabetic pregnancies (3, 5, 6), increasing progressively during the last months of gestation (6, 7). Clinical and experimental studies suggest that unexpected late-pregnancy stillbirths in diabetic pregnancies are in most cases caused by fetal hypoxia (8). Macrosomic or growth-restricted fetuses, in particular, are at high risk, but stillbirths are also seen in fetuses within normal birth weight percentiles (7–9). Although the exact feto-placental molecular mechanisms behind fetal hypoxia in diabetic pregnancies are unknown, fetal hyperglycemia and hyperinsulinemia appear to be key players in its pathogenesis (7, 8). In line with this, maternal BMI and glycemic control have been identified as important modifiable risk factors of stillbirth in pregnancies complicated by maternal diabetes (7).

Erythropoietin (EPO) regulates erythropoiesis in the fetus similarly as in adults and hypoxia is a major stimulus for its synthesis. Before 30 weeks’ gestation, liver is the primary site of fetal EPO synthesis, after which a gradual transition to renal EPO production occurs (10, 11). During normal oxygenation at term, fetal EPO production takes place mainly in the kidneys (11). Since EPO is not stored and does not cross the placenta, fetal plasma EPO levels indicate fetal EPO production and elimination during normoxia. When the fetus suffers from prolonged hypoxia, both cord blood and amniotic fluid EPO (AF EPO) concentrations increase exponentially (12). AF EPO concentrations have been demonstrated to correlate well with both low (normal) or high (abnormal) simultaneously obtained fetal plasma EPO concentrations (13). Moreover, in our previous study, we have shown that AF EPO in type 1 diabetic pregnancies correlates negatively with umbilical artery pH, pO2 and neonatal lowest blood glucose level, and positively with umbilical artery pCO2 and last maternal HbA_1c_ value before delivery (14). We have also observed a U-shaped correlation between AF EPO and birthweight z score (14).

In addition to its well-established role in erythropoiesis, more recently, EPO has been identified to function as a tissue protective molecule, exerting anti-inflammatory, anti-apoptotic, antioxidant and neurotrophic effects (15, 16). Interestingly, available data indicates that the EPO concentrations required to trigger these tissue-protective effects, mediated by specific EPO receptor isoforms, are ~100–1 000 times higher than the concentrations needed for the regulation erythropoiesis (15, 16). Considering this background, it has been speculated that feto-placental EPO production increases drastically in fetal chronic hypoxia to protect vital organs (e.g. central nervous system) (17, 18). Although the organ that secretes the high concentrations of EPO into the fetal circulation in hypoxia remains elusive, the placenta is a strong candidate as the source tissue (19), based on studies in sheep (20) and in monochorionic twin pregnancies (21).

Experimental studies have shown that fetal serum/plasma EPO levels increase ~3-4 hours [26, 27] and AF EPO ~6 hours after the onset of moderate to severe hypoxemia [27]. Since a minimum of several hours of hypoxia is needed to trigger the elevation of AF EPO, its measurement can be used to diagnose fetal chronic hypoxia antenatally (14, 22). This method has been in clinical use at Helsinki University Hospital (HUH), Department of Obstetrics and Gynecology, since 1995, to optimize the timing of delivery.

To the best of our knowledge, no previous studies have investigated whether fetal hypoxia in type 1 diabetic pregnancies predisposes the offspring to adverse metabolic health outcomes in later life. In this study, we aimed to compare indicators of metabolic health between 1) young adult offspring of women with type 1 diabetes (OT1D) with elevated antenatal AF EPO concentrations indicating fetal hypoxia; 2) OT1D with low antenatal AF EPO concentrations; and 3) offspring born from uncomplicated pregnancies to non-diabetic mothers.

## Materials and Methods

Between 1^st^ September 1995 and 31^st^ December 2000, a total of 331 women with type 1 diabetes gave birth at the Department of Obstetrics and Gynecology, University Hospital, Helsinki. Concerning these childbirths, the perinatal outcomes of 156 singleton pregnancies with measured AF EPO levels have been published previously (14). These women with type 1 diabetes were delivered by caesarean section before labor and had AF sampled for EPO measurement within two days before delivery. Normal AF EPO concentrations (<14.0 mU/ml) occurred in 49.0% (76/156) of the cases and abnormal (≥14.0 mU/ml) in 51.0% (80/156) (14).

In the present study, the offspring from these 156 pregnancies were invited to participate in a follow-up including a clinical examination, laboratory tests and questionnaires at young adult age (18-23 years) between 1^st^ March and 20^th^ November 2019. A flow chart depicting the formation of the study population is shown in **Supplementary Figure 1**. In total, 58/156 (37.2%) offspring of women with type 1 diabetes (OT1D) agreed to participate. Similarly to our previous study (14), AF EPO <14.0 mU/l within 2 days before birth was defined as normal. In total, 29 OTID fulfilled this criterion and were categorized into the low EPO (L-EPO) group. The remaining 29 OT1D had AF EPO ≥14.0 mU/ml, defined as fetal hypoxia, and were categorized into the high EPO (H-EPO) group.

A control group of 442 mother-offspring pairs without maternal diabetes and normal, uncomplicated pregnancies, matched for offspring date and place of birth, were identified from the Finnish Medical Birth Register. Of the invited control offspring, a total of 86/442 (19.5%) young adults agreed to take part in the study (**Supplementary Figure 1**).

All study participants provided a written informed consent. The study protocol was approved by the Ethics Committee of the Hospital District of Helsinki and Uusimaa (HUS/898/2017, 14 December 2017) and the study was implemented in accordance with the Declaration of Helsinki.

### Collection of Maternal and Perinatal Data

Regarding type 1 diabetic pregnancies, information on maternal characteristics as well as obstetric and perinatal outcomes were collected from maternity care and hospital records as described previously (2, 14). Glycated hemoglobin A_1c_ (HbA_1c_) was assessed by a high-performance liquid chromatography method (Diamat, Bio-Rad Laboratories, Hercules, CA, USA). Three HbA_1c_ values were used for the purpose of this study: the first one in the first trimester, one value (or the mean if more than one was assessed) measured between 18 + 0 to 22 + 0 weeks’ gestation, and the last value before delivery. The second highest systolic and diastolic blood pressure (BP) values in each trimester were recorded, ensuring that the BP levels used to determine the diagnosis of hypertensive disorders were based on repeated measurements exceeding the diagnostic thresholds. Pre-eclampsia was defined as systolic BP ≥140 mmHg and/or diastolic BP ≥90 mmHg occurring after 20 weeks’ gestation in a previously normotensive woman, combined with new-onset proteinuria of ≥0.3 g/24h (23). Gestational hypertension was defined similarly, but without the presence of proteinuria. Chronic hypertension was defined as BP ≥140 mmHg in the first trimester or diagnosis of chronic hypertension before pregnancy. Umbilical artery samples were analysed at birth for pH using Ciba-Corning, Rapidlab 800 (Bayer Siemens) and ABL (Radiometer) pH/blood gas analysers. AF EPO concentrations were measured until 31 July 1998 in duplicate by RIA (EPO-Trac, Incstar, Stillwater, Minn., USA) and, thereafter, by a chemiluminescent immunological method (Immulite EPO Assay; Diagnostic Products, Los Angeles, Calif., USA). Similar results were obtained with these two methods (14). The assays were standardised according to the World Health Organization’s Second International Reference Preparation for EPO. Birth weights >2.0 SD units (>97.7^th^ percentile) were defined as macrosomia and those <-2.0 SD units (<2.3^th^ percentile) as small-for-gestational age (SGA) using a Finnish standard population (24). Neonatal hypoglycemia was defined as blood glucose <2.6 mmol/l in the early neonatal period.

Regarding the control mother-offspring pairs, information on maternal age at delivery, delivery mode, birth weight, and umbilical artery pH values were collected from the Finnish Medical Birth Register. Offspring relative birthweight was calculated as z-scores (according to sex and gestational age) (24).

### Follow-Up at Young Adult Age

The weight and body composition of all study participants were measured utilizing a bioimpedance device (InBody 3.0 eight-polar tactile electrode system, Biospace Co, Ltd, Seoul, Korea). The instrument estimates lean body mass and body fat mass by segmental multi-frequency (5, 50, 250, and 500 kHz) analysis. The measurements were made with the subject standing in light indoor clothing on the foot electrodes of the platform and gripping the palm and thumb electrodes. Body fat percentage was calculated as fat mass (kg) divided by body weight (kg) converted to percent (%). Weight was measured to an accuracy of 0.1 kg. Height was measured without shoes on by a measuring tape against a wall to an accuracy of 0.1 cm. BMI was calculated as body weight divided by height squared (kg/m^2^). Waist circumference (cm) was measured midway between the anterior superior iliac spine and lower edge of the rib cage in a relaxed standing posture. After at least 15 minutes of rest, BP was measured from the right arm, in a sitting position, using a cuff size of 22 cm x 42 cm. The BP measurements were repeated three times with a minimum pause of one minute between the measurements. The mean of the three BP values is reported.

Venous blood samples were taken in the morning, after ten hours of fasting, in a sitting position, for the analysis of lipids (total cholesterol, HDL, LDL, triglyceride), high-sensitivity CRP (hs-CRP), and HbA_1c_. The participants then underwent a standard 2-hour OGTT with 75 g of glucose according to the WHO 1999 guidelines. A blood sample for plasma glucose (PG) and serum insulin analysis was drawn in fasting stage. At this point, two OT1D (one in each EPO group) were diagnosed with overt diabetes based on their fasting plasma glucose value >7.0 mmol/L and excluded from all further analyses. The participants without diabetes ingested a 300 mL solution containing 75 g anhydrous glucose and 1.6 g citric acid. At 1h and 2h timepoints after the ingestion of this solution, blood samples were drawn for the glucose and insulin measurements. All laboratory analyses were carried out utilizing routine, standardized methods at the Laboratory of Helsinki University Hospital (HUSLAB). Cholesterol, triglyceride, plasma glucose and HbA_1c_ concentrations were assessed with a photometric enzymatic method and hsCRP with an immunoturbidimetric method utilizing an Atellica^®^ CH 930 Analyzer (Siemens Healthineers, Tarrytown, USA). Serum insulin was assessed with an immunochemiluminometric method utilizing a Liaison^®^ XL Analyzer (DiaSorin SpA, Saluggia VC, Italy). Homeostasis model assessment - insulin resistance (HOMA-IR) index was calculated with the formula FPG (mmol/l) x fasting serum insulin (mU/l) divided by 22.5.

In addition to metabolic parameters, leisure-time physical activity (LTPA) was assessed utilizing the validated Kuopio Ischemic Heart Disease Risk Factor Study 12‐month LTPA recall questionnaire (25). The subjects were asked to fill in the type, frequency and average duration of the different types of physical activities performed each month for the past 12-months. For each activity, a metabolic equivalent of task (MET, 1 MET = 3.5 ml O2 kg−1 min−1 or 1 kcal kg−1 h−1) value was assigned based on the compendium of physical activities (26). The volume of LTPA was calculated by the sum of MET-values multiplied by the frequency and duration of activities. The total volume of LTPA was expressed as MET-hours (METh) per week.

### Statistical Methods

Descriptive statistics are presented as means with standard deviation (SD), as medians with interquartile range (IQR) or as counts with percentages. Statistical comparisons between groups were done using the t-test, analysis of variances or the chi-square test. The relationships between AF EPO and 2-hour post-load glucose and insulin concentrations were derived from linear regression models. The bootstrap method was used when the theoretical distribution of the test statistics was unknown or in the case of violation of the assumptions (e.g. non-normality). Hommel’s adjustment was applied where appropriate to correct levels of significance for multiple testing (*post hoc*). Crude and partial correlation coefficients with 95% confidence intervals (CI) were calculated by using the Pearson method. The normality of variables was evaluated graphically and by the Shapiro–Wilk W test. All statistical analyses were carried out with Stata, version 15.1 (StataCorp, College Station, TX, USA).

## Results

Maternal and perinatal characteristics of the mother-offspring pairs affected by type 1 diabetes, categorized based on prenatal AF EPO concentrations, and their matched controls, are displayed in **Table 1**. As expected, gestational age at birth was lower and birth weight z-scores higher in the L-EPO and H-EPO groups compared to the controls. No statistically significant differences were observed between the L-EPO and H-EPO groups with respect to these variables, nor concerning maternal age, pre-gestational BMI, diabetes duration, HbA_1c_ concentrations during pregnancy or hypertensive disorders. The median (IQR) AF EPO concentrations in the total cohort of OT1D and in the L-EPO and H-EPO groups were 14.2 (10.0/18.0), 10.0 (8.0/12.0) and 18.0 (15.0/40.4) mU/mL, respectively.

**Table 1:** Maternal and perinatal characteristics of mother-offspring pairs affected by maternal type 1 diabetes and matched control (C) offspring.

Maternal and perinatal characteristics	Controls (C) *n*=86	Low EPO (L) *n*=28	High EPO (H) *n*=28	*p* value (multiple comparisons*)
Maternal age (years)	21.1 (1.5)	20.9 (1.7)	20.9 (1.4)	0.70
Pre-gestational BMI (kg/m^2^)	–	23.2 (2.6)	23.7 (3.0)	0.49
Age at diabetes diagnosis (years)	–	11.4 (5.8)	13.9 (8.3)	0.19
Diabetes duration (years)	–	9.5 (5.7)	7.0 (8.7)	0.21
First trimester HbA_1c_ (%; mmol/mol)	–	7.3 (1.1); 56 (8.4)	7.5 (1.1); 58 (8.5)	0.40
Mid-trimester HbA_1c_ (%; mmol/mol)	–	6.8 (1.0); 51 (7.5)	6.6 (0.9); 49 (6.7)	0.59
Last HbA_1c_ before delivery (%; mmol/mol)	–	6.6 (1.1); 49 (8.2)	6.9 (1.1); 52 (8.3)	0.41
Third trimester systolic blood pressure (mmHg)	–	141 (26)	144 (25)	0.62
Third trimester diastolic blood pressure (mmHg)	–	84 (12)	88 (14)	0.21
Gestational hypertension	0 (0)	5 (18)	7 (25)	0.52
Preeclampsia	0 (0)	3 (11)	9 (32)	0.10
Cesarean section	22 (26)	28 (100)	28 (100)	<0.001
Gestational age at birth (weeks)	39.9 (1.6)	37.1 (1.3)	36.4 (1.8)	<0.001 (C/L, C/H)
Fetal sex (female/male)	57 (66)/29 (34)	17 (61)/11 (39)	19 (68)/9 (32)	0.83
Birth weight (BW) z-score (SD units)	0.13 (0.91)	0.87 (1.94)	1.43 (2.15)	<0.001 (C/L, C/H)
Fetal macrosomia (BW z-score >2.0 SD units)	5 (6)	8 (29)	8 (29)	<0.001 (C/L, C/H)
Small-for-gestational age (BW z-score < -2.0 SD units)	0 (0)	1 (4)	1 (4)	0.21
Umbilical artery pH	7.27 (0.08)	7.25 (0.04)	7.25 (0.04)	0.12
Neonatal hypoglycemia	–	10 (36)	15 (54)	0.18

Values are mean (SD) or frequencies (%).

*Hommel’s multiple comparison procedure was used to correct significance levels for post hoc testing (p<0.05 for pairwise comparisons indicated in parentheses). The offspring of women with type 1 diabetes were categorized into those with low (<14.0 mU/L; L) and high (≥14.0 mU/L; H) amniotic fluid erythropoietin (EPO) concentrations within 2 days before birth.

**Table 2** shows the metabolic parameters of OT1D and control offspring at young adult age. The parameters which are known to be significantly affected by sex are shown separately for female and male offspring. The BMI of OT1D in the H-EPO group was higher than that of the L-EPO offspring. Women in the H-EPO group had a higher waist circumference compared to those in the L-EPO group or the control women, but no differences were noted between the male offspring groups. Body fat percentage was also higher among the H-EPO women compared to the control women. Due to this sexual dimorphism within the OT1D groups with regards to adult body composition, we also examined interactions between maternal and perinatal characteristics, AF EPO levels and fetal sex, but no significant interactions were found (**Supplementary Table 1**).

**Table 2:** Metabolic characteristics and leisure-time physical activity (LTPA) volumes in young adult offspring born from pregnancies affected by maternal type 1 diabetes and in control (C) offspring born from non-diabetic pregnancies matched according to age and place of birth.

Offspring characteristics at young adult age	Controls (C)	Low EPO (L)	High EPO (H)	*p* value
	N=86	n=28	n=28	(multiple comparisons*)
Age (years)	21.1 (1.5)	20.9 (1.7)	20.9 (1.4)	0.70
BMI (kg/m^2^)	24.3 (5.3)	22.4 (3.1)	26.3 (6.3)	0.016 (L/H)
Weight (kg)				
Women	68 (17)	60 (9)	75 (18)	0.28
Men	80 (14)	78 (12)	75 (20)	0.38
Height (cm)				
Women	167 (6)	166 (6)	166 (6)	0.56
Men	182 (8)	183 (6)	176 (6)	0.065
Waist circumference (cm)				
Women	78 (13)	73 (6)	87 (15)	0.003 (C/H, L/H)
Men	86 (10)	85 (9)	86 (17)	0.94
Body fat percentage (%)				
Women	30.3 (9.5)	28.5 (5.9)	36.9 (7.3)	0.030 (C/H)
Men	18.4 (8.0)	17.5 (8.3)	20.5 (14.1)	0.66
Systolic blood pressure (mmHg)	118 (10)	117 (13)	117 (13)	0.84
Diastolic blood pressure (mmHg)	74 (7)	73 (10)	74 (10)	0.82
hs-CRP (mg/L)	2.0 (2.5)	1.3 (1.6)	1.6 (2.0)	0.26
Total cholesterol (mmol/L)	4.31 (0.65)	4.42 (0.69)	4.45 (0.70)	0.55
LDL Cholesterol (mmol/L)	2.56 (0.66)	2.50 (0.70)	2.75 (0.70)	0.33
HDL Cholesterol (mmol/L)				
Women	1.64 (0.38)	1.83 (0.40)	1.51 (0.33)	0.44
Men	1.38 (0.30)	1.52 (0.34)	1.48 (0.31)	0.25
Triglycerides (mmol/L)	0.98 (0.46)	0.89 (0.51)	1.02 (0.67)	0.62
Fasting plasma glucose (mmol/L)	5.40 (0.42)	5.39 (0.33)	5.40 (0.42)	0.92
2-hour plasma glucose (mmol/L)	5.67 (1.48)	5.21 (1.10)	6.50 (2.11)	0.009 (C/H, L/H)
Fasting serum insulin (mmol/L)	10.8 (6.9)	9.4 (4.5)	13.4 (13.7)	0.20
2-hour serum insulin (mmol/L)	59.4 (48.8)	47.2 (29.4)	82.4 (99.7)	0.10
HOMA-IR	2.64 (1.86)	2.28 (1.13)	3.35 (3.55)	0.17
HbA_1c_ (mmol/mol)	32.6 (2.2)	31.8 (2.0)	33.0 (3.2)	0.16
Total LTPA, METh/wk	37.7 (44.3)	26.0 (14.4)	32.0 (38.3)	0.10

Values are mean (SD) or frequencies (%).

Hs-CRP, High-sensitivity c-reactive protein; MET, Metabolic Equivalent of Task.

*Hommel’s multiple comparison procedure was used to correct significance levels for post hoc testing (p<0.05 for pairwise comparisons indicated in parentheses). The offspring of women with type 1 diabetes were categorized into those with low (<14.0 mU/L; L) and abnormally high (≥14.0 mU/L; H) amniotic fluid erythropoietin (EPO) levels within two days before birth.

**Figure 1:**
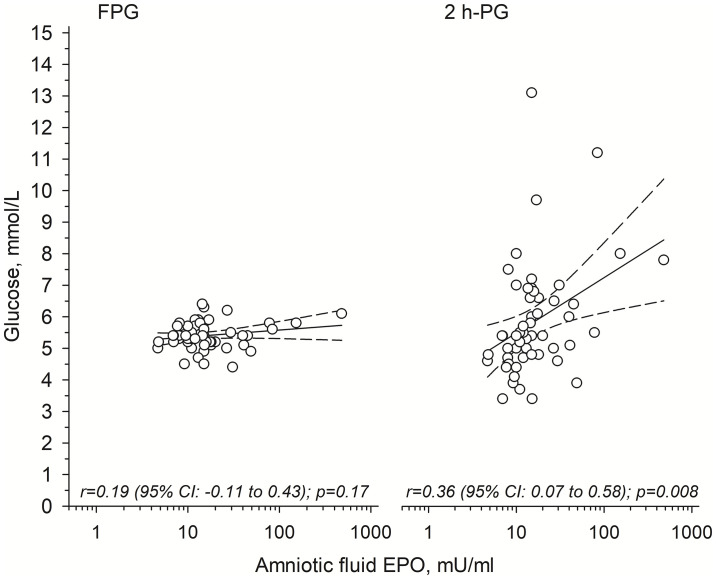
Relationships between antenatally measured amniotic fluid erythropoietin (EPO) and 1) fasting plasma glucose (FPG; *left panel*); and 2) 2-hour post-load plasma glucose (2h-PG; *right panel*) concentrations in young adult offspring of mothers with type 1 diabetes. Solid line shows the regression line and dashed line shows the 95% confidence intervals.

In the OGTTs, fasting PG concentrations were similar in all groups (**Table 2**) and no correlation between antenatal AF EPO and fasting PG at young adult age was observed [r=0.19 (95% CI: -0.11 to 0.43)] (**Figure 1**, *left panel*). In contrast, 2-hour post-load PG concentrations were higher in the H-EPO group as compared to the L-EPO and control offspring (**Table 2**). Moreover, a positive correlation was detected between AF EPO and 2-hour post-load PG concentrations among the OT1D (**Figure 1**, *right panel*), even after adjustment for maternal pre-gestational BMI, offspring relative birth weight, gestational age at birth, and offspring young adult BMI [non-adjusted r=0.36 (95% CI: 0.07 to 0.58); adjusted r=0.35 (95% CI: 0.07 to 0.62)].

Fasting or 2-hour serum insulin concentrations did not differ between the OT1D groups or compared to the controls. However, among the OT1D, AF EPO correlated positively with 2-hour post-load serum insulin [non-adjusted r=0.42 (95% CI: 0.06 to 0.61)], and the correlation was strengthened by adjustment for maternal pre-gestational BMI, offspring relative birth weight, gestational age at birth, and offspring young adult BMI (adjusted r=0.44 (95% CI: 0.14 to 0.69)]. No significant correlation between AF EPO and fasting insulin concentration was detected.

There were no differences in BP levels, hsCRP, triglyceride or cholesterol levels, HbA_1c_ or LTPA volumes between the control, L-EPO and H-EPO offspring in early adulthood. Again, because of the sexual dimorphism among the OT1D with respect to waist circumference and body fat percentage, LTPA volumes in the OT1D groups were also categorized by sex. In this analysis, we observed similar mean (SD) LTPA volumes (METh/wk) among the females [25.9 (15.2)] *vs.* males [26.2 (13.6)] in the L-EPO group, but higher LTPA volumes in the females [40.4 (43.2)] *vs.* males [14.3 (15.0)] in the H-EPO group (main effects EPO categories p= 0.84, offspring sex p=0.036, and their interaction p=0.034).

## Discussion

The main finding of the present study is that elevated AF EPO concentrations within two days before birth, suggesting intrauterine hypoxia, were associated with higher 2-hour post-load PG concentrations in young adult OT1D, compared to OT1D who had normal AF EPO concentrations and control offspring born from non-diabetic pregnancies. This observation was further strengthened by the positive correlations between prenatal AF EPO concentrations and post-load PG and insulin levels in OT1D in early adulthood, even when adjusted for confounders such as maternal BMI, offspring relative birth weight and offspring adult BMI. Additionally, OT1D with elevated AF EPO levels in late pregnancy were characterized by adiposity, with higher BMI recorded in both male and female OT1D, and higher waist circumference and body fat percentage in female OT1D, compared to OT1D with low AF EPO levels or controls.

Evidence linking maternal diabetes during pregnancy with adverse cardiometabolic outcomes in the offspring is particularly abundant concerning gestational and type 2 diabetes, but fewer studies examining solely OT1D have been performed, especially with follow up extending to adulthood (27, 28). To the best of our knowledge, we are the first to examine associations between fetal hypoxia, as evidenced by AF EPO concentrations, and long-term offspring health outcomes in pregnancies affected by diabetes. Other strengths of the present study include detailed characterization of type 1 diabetes pregnancies and the availability of high-quality national health register data that enabled the matching of control participants according to age and place of birth. On the other hand, a clear limitation of our study is that less complete maternal and perinatal data was available on the controls. Maternal pre-gestational BMI is a major determinant of offspring adiposity (29), but this variable was not yet consistently registered at the time the control offspring were born. Maternal gestational weight gain or breastfeeding could also have impacts on offspring metabolic health in adulthood, but we did not have access to this data. Further, due to the study design, prenatal AF EPO values were not available on the control participants; however, the likelihood of abnormal values in uncomplicated pregnancies of healthy women is very low (30, 31). Finally, many of the invited offspring did not agree to participate in the study at young adult age, which reduced the available sample size, and may have introduced some selection bias.

Previous studies have reported higher BMI levels among OT1D, compared to individuals born from non-diabetic pregnancies, in childhood (32–34), adolescence (34–37) and adulthood (27). Although the exact mechanisms are still unclear, landmark studies in Pima Indians (38) and European populations (34, 39) support the view that diabetic intrauterine conditions contribute to increased adiposity in adult offspring. Across the maternal glucose spectrum including the normoglycemic range, higher glucose concentrations associate with a higher risk of childhood adiposity, independent of maternal pre-pregnancy BMI and family history of diabetes (29, 40). Interestingly, in our cohort, BMI at adult age was increased only in the H-EPO group in comparison to the L-EPO group, which had the lowest mean BMI. Differences in maternal pre-pregnancy BMI, gestational age at birth, delivery mode or offspring physical activity at young adult age do not appear to explain this, since these were comparable between the L- and H-EPO groups. However, the H-EPO offspring group had the highest relative birth weight and neonatal hypoglycemia frequency, even though these differences between the L- and H-EPO groups did not reach statistical significance. Therefore, the H-EPO offspring may have been more hyperinsulinemic, but unfortunately umbilical cord insulin or C-peptide levels were not collected in the original study (14). It is also possible that specific feto-placental alterations or characteristics that predispose to fetal metabolic dyshomeostasis, and hence, to chronic fetal hypoxia in late pregnancy, program offspring energy metabolism towards proneness to adiposity in later life. Lindsay et al. has previously observed a positive correlation between cord blood hematocrit at birth and BMI at age 7 in OT1D, also implicating a possible link between late pregnancy fetal hypoxia and later offspring adiposity (41).

Increasing evidence suggests differences in the growth strategies of male and female fetuses and their vulnerability to stress and maternal metabolic derangements (7, 42). Although a recent study found few differences in the perinatal outcomes of type 1 diabetic pregnancies due to fetal sex (43), Lohse et al. observed increased total body fat percentage and reduced adiponectin levels in female, but not in male, adolescent OT1D, compared to offspring born from non-diabetic pregnancies (37). These results are congruent with our findings of increased body fat percentage and waist circumference in the female OT1D. However, a new observation is that this sex difference was apparent only in the H-EPO offspring, i.e. those who have likely been affected by the most severe imbalance of feto-placental metabolism in late pregnancy.

Robust evidence has established that exposure to any type of maternal diabetes increases the offspring’s risk of prediabetes and type 2 diabetes. The two cases (3.4%) of overt diabetes identified among the OT1D, and the higher 2-hour post-load glucose concentrations in the H-EPO group, are in line with previous studies in children (34), adolescents (36, 37) and adults (28, 44) demonstrating various degrees of derangement in glucose metabolism in OT1D, with elevated post-load PG and insulin concentrations being a typical finding. The HOMA-IR values, showing a trend towards higher values in the H-EPO group, are also in agreement with this picture, although statistical significance was not reached. However, surprisingly, paralleling our findings with respect to body composition, OT1D in the L-EPO group presented with a glycemic profile comparable to that of the controls. This could indicate better feto-placental metabolic flexibility, or protective mechanisms, in the OT1D who avoided the development of late-pregnancy fetal hypoxia, with potential positive metabolic programming effects extending into adulthood. Notably, despite the central adiposity in the female offspring of the H-EPO group, no sex differences were observed in the parameters reflecting glucose metabolism. It is possible that the higher LTPA volumes in the female H-EPO offspring may have had compensatory impacts on insulin sensitivity.

Maternal diabetes can promote the development of fetal hypoxia *via* multiple mechanisms (45) and these, or the effects of hypoxia itself, could contribute to alterations of glucose metabolism in the H-EPO group. Strong evidence from animal and human studies supports the hypothesis that fetal hyperglycemia and -insulinemia are central factors in the pathogenesis of diabetes-associated fetal hypoxia, although the exact cellular mechanisms within the feto-placental unit remain unclear (8). In accordance with this, fetal EPO levels have been shown to correlate with maternal glycemic control (14) as well as AF and fetal plasma insulin levels (46, 47). While no statistically significant differences in maternal glycemic control were noted between the L- and H-EPO groups, both early- and late-pregnancy HbA_1c_ displayed a tendency toward higher levels in the H-EPO pregnancies. It is also possible that “time-in-target” or glycemic variability has been less optimal in this group. Considering the positive association between fetal insulin levels and the incidence of later impaired glucose tolerance in the offspring (34, 35), the correlation between AF EPO and offspring post-load PG concentrations in adulthood could be mediated by fetal hyperinsulinemia. It is also possible that fetal programming mechanisms, feto-placental metabolic changes, or tissue damage directly linked to fetal hypoxia are involved. The role of mechanistic pathways other than those related to fetal hyperinsulinemia is supported by studies in non-diabetic conditions – such as fetal growth restriction, hypertensive disorders and post-term pregnancies – that are also characterized by increased risks of fetal hypoxia, high EPO and stillbirths (7, 22, 48) as well as later adverse metabolic health outcomes in the offspring (49–51).

Almost identical BP levels were observed in the three groups of young adults examined. In contrast, Clausen et al. observed an increased frequency of systolic hypertension in adult OT1D (27). Also, a recent large population-based study reported maternal diabetes to be associated with elevated rates of early-onset cardiovascular disease, including hypertensive disorders, in the offspring, from childhood to early adulthood (52). It is possible that our sample size was insufficient to detect slight differences in this parameter, especially considering that overt hypertension generally develops at older age.

## Conclusions

In conclusion, the present study provides novel evidence on the potential role of late-pregnancy fetal hypoxia in the programming of adiposity and altered glucose metabolism in adult OT1D. Our findings give yet another reason to strive for normal pre-pregnancy BMI, meticulous glycemic control, and careful timing of delivery in type 1 diabetic pregnancies, to reduce the risk of fetal hypoxia. Furthermore, our results underscore the importance of utilizing maternal pregnancy history in the identification of individuals at the highest risk of metabolic morbidity.

## Data Availability Statement

The datasets presented in this article are not readily available because data cannot be shared for both legal and ethical reasons. Requests to access the datasets should be directed to merja.k.laine@helsinki.fi.

## Ethics Statement

The studies involving human participants were reviewed and approved by the Ethics Committee of the Hospital District of Helsinki and Uusimaa (HUS/898/2017, 14 December 2017). The patients/participants provided their written informed consent to participate in this study.

## Author Contributions

MK, KT, JE, and ML made substantial contributions to the conception and design of this study, as well as the acquisition, analysis, and interpretation of data. NW calculated LTPA volumes. HK performed all statistical analyses. MK wrote the article in collaboration with KT. All authors were involved in revising the article critically for important intellectual content. All authors approved the final version of the article to be published.

## Funding

This study was funded by Finska Läkaresällskapet and by State Funding for University-Level Health Research awarded to the Hospital District of Helsinki and Uusimaa, Finland. MK was funded by the Finnish Cultural Foundation (*via* the Finnish Foundations’ Postdoc Pool), Biomedicum Helsinki Research Foundation, Finnish Medical Foundation, Juho Vainio Foundation, and Vyborg Tuberculosis Foundation. The study funders were not involved in the design of the study; the collection, analysis, and interpretation of data; or writing the report; and did not impose any restrictions regarding the publication of the report

## Conflict of Interest

The authors declare that the research was conducted in the absence of any commercial or financial relationships that could be construed as a potential conflict of interest.

## Publisher’s Note

All claims expressed in this article are solely those of the authors and do not necessarily represent those of their affiliated organizations, or those of the publisher, the editors and the reviewers. Any product that may be evaluated in this article, or claim that may be made by its manufacturer, is not guaranteed or endorsed by the publisher.

## Supplementary Material

Supplementary Figure 1 A diagram representing the formation of the study population. Offspring of women with type 1 diabetes (OT1D); amniotic fluid erythropoietin (AF EPO); OT1D with low AF EPO <14.0 mU/ml (L-EPO); OT1D with high AF EPO ≥ 14.0 mU/ml (L-EPO).

## Abbreviations

AF EPO, amniotic fluid erythropoietin; FPG, fasting plasma glucose; HbA_1c_, glycated hemoglobin A_1c_; HOMA-IR, homeostatic model of assessment – insulin resistance; hs-CRP, high-sensitivity c-reactive protein; HUH, Helsinki University Hospital; LTPA, leisure-time physical activity; OT1D, offspring of women with type 1 diabetes; PG, plasma glucose; 2h-PG, 2-hour post-load plasma glucose.
