# Supplementary material for: Late-Pregnancy Fetal Hypoxia Is Associated With Altered Glucose Metabolism and Adiposity in Young Adult Offspring of Women With Type 1 Diabetes
Source: Front Endocrinol (Lausanne). 2021 Oct 27;12:738570. doi: 10.3389/fendo.2021.738570 (PMC8578885; doi:10.3389/fendo.2021.738570)
Supplement: Supplementary file 1 [file DataSheet_1.pdf]

Supplemental Material

Supplementary Table 1. Comparison of maternal and perinatal characteristics by fetal sex in pregnancies of women with type 1 diabetes with low (<14.0 mU/L; L-EPO) and high (≥14.0 mU/L; H-EPO) amniotic fluid erythropoietin (EPO) concentrations measured within 2 days before delivery.

Maternal or perinatal characteristics	L-EPO		H-EPO		P-value		
	Female N=17	Male N=11	Female N=19	Male N=9	EPO	Fetal sex	Interaction
Maternal pre-pregancy BMI (kg/m^2)	23.8 (2.8)	22.3 (2.1)	23.8 (2.5)	23.6 (4.2)	0.41	0.28	0.39
First trimester HbA_{1c} (%; mmol/mol)	7.4 (1.2); 58 (9.4)	7.1 (0.9); 54 (6.8)	7.7 (1.1); 61 (8.7)	7.1 (1.1); 54 (8.4)	0.60	0.092	0.61
Mid-trimester HbA_{1c} (%; mmol/mol)	6.9 (1.1); 52 (8.3)	6.6 (0.6); 49 (4.5)	6.7 (0.8); 50 (5.9)	6.4 (1.1); 46 (7.9)	0.54	0.22	0.99
Last HbA_{1c} before delivery (%; mmol/mol)	6.7 (1.4); 50 (10.4)	6.6 (0.7); 49 (5.2)	6.8 (0.9); 51 (6.8)	7.0 (1.5); 53 (11.4)	0.38	0.90	0.68
Third trimester systolic BP (mmHg)	142 (30)	139 (20)	140 (24)	154 (24)	0.39	0.44	0.26
Third trimester diastolic BP (mmHg)	84 (13)	84 (12)	84 (13)	96 (11)	0.068	0.090	0.088
Gestational hypertension	2 (12)	3 (27)	3 (16)	4 (44)	0.42	0.069	0.76
Preeclampsia	2 (12)	1 (9)	6 (32)	3 (33)	0.066	0.89	0.81
Gestational age at birth (weeks)	37.1 (1.6)	37.1 (0.7)	36.4 (1.8)	36.5 (2.0)	0.17	0.99	0.90
Preterm birth (<37 weeks' gestation)	7 (41)	4 (36)	11 (58)	4 (44)	0.38	0.51	0.77
Relative birth weight (BW) (SD units)	0.73 (2.16)	1.09 (1.61)	1.67 (2.25)	0.92 (1.96)	0.51	0.74	0.34
Fetal macrosomia (BW z-score > +2.0 SD units)	5 (29)	3 (27)	7 (37)	1 (11)	0.74	0.28	0.36

Values are mean (SD) or frequencies (%)
