# Supplementary figures and images for: Late-Pregnancy Fetal Hypoxia Is Associated With Altered Glucose Metabolism and Adiposity in Young Adult Offspring of Women With Type 1 Diabetes
Source: Front Endocrinol (Lausanne). 2021 Oct 27;12:738570. doi: 10.3389/fendo.2021.738570 (PMC8578885; doi:10.3389/fendo.2021.738570)

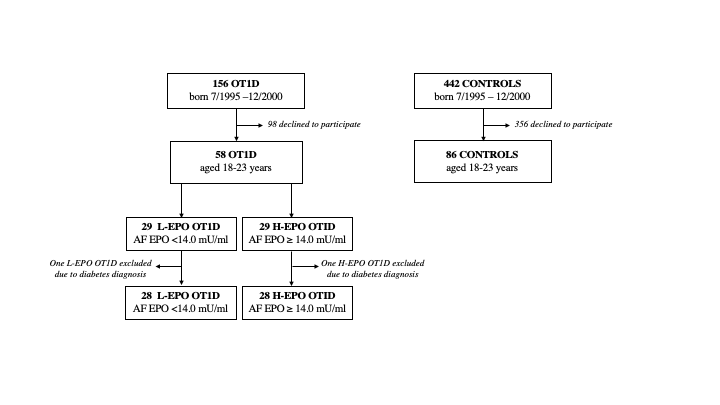

Supplement: Supplementary Figure 1 — A diagram representing the formation of the study population. Offspring of women with type 1 diabetes (OT1D); amniotic fluid erythropoietin (AF EPO); OT1D with low AF EPO <14.0 mU/ml (L-EPO); OT1D with high AF EPO ≥ 14.0 mU/ml (L-EPO). [file Image_1.tiff]
